# Identifying a *BRCA2* c.5722_5723del mutation in a Han-Chinese family with breast cancer

**DOI:** 10.1042/BSR20182471

**Published:** 2019-04-30

**Authors:** Yi Guo, Peng Wang, Xiaorong Li, Shaihong Zhu, Hongbo Xu, Shizhou Li, Hao Deng, Lamei Yuan

**Affiliations:** 1 Center for Experimental Medicine, The Third Xiangya Hospital, Central South University, Changsha, China; 2 Department of Medical Information, School of Life Sciences, Central South University, Changsha, Hunan, China; 3 Department of Gastrointestinal Surgery, The Third Xiangya Hospital, Central South University, Changsha, China; 4 Department of General Surgery, The Third Xiangya Hospital, Central South University, Changsha, China; 5 Department of Neurology, The Third Xiangya Hospital, Central South University, Changsha, China

**Keywords:** breast cancer, BRCA2, genetic, homologous recombination, mutation

## Abstract

Breast cancer (BC) is the most common female cancer found worldwide. It is responsible for 25% of all cancer patients in females. Hereditary BC accounts for about 5–10% of all BC cases. The breast cancer 1 gene (*BRCA1*) and the breast cancer 2 gene (*BRCA2*) are the two most-studied BC susceptibility genes. Genetic testing for disease-causing mutations in *BRCA1, BRCA2*, and other BC susceptibility genes is strongly recommended for members of families having a BC family history. The present study found a heterozygous c.5722_5723del mutation in the *BRCA2* exon 11 of a large Han-Chinese BC family using whole exome sequencing and Sanger sequencing. It may cause DNA double-strand breaks repair dysfunction by disturbing homologous recombination, further resulting in BC. The study findings may help supplement and further improve genetic testing strategies and BC risk estimation methodologies in China.

## Introduction

Breast cancer (BC) is the primary cause of cancer-related deaths and the most frequent female cancer worldwide. It is responsible for 15% of all cancer deaths and 25% of all cancer patients in females [1]. According to the GLOBOCAN 2012 database, BC incidence and mortality in East Asia are 0.027% and 0.0061%, respectively [1]. Incidence rates have been rising. Hereditary BC accounts for 5–10% of all BC cases [2]. A variety of genes and mutations are related to BC. Germline mutations in the breast cancer 1 gene (*BRCA1*, OMIM 113705) and the breast cancer 2 gene (*BRCA2*, OMIM 600185) are the most-studied genetic alterations causing autosomal dominant familial breast-ovarian cancer (BOC) [3–5]. The estimated penetrance of *BRCA1* mutation carriers by age 70 was 65% for BC and 45% for ovarian cancer, and the corresponding rates of *BRCA2* mutation carriers were 45% and 11% [6]. Disease-causing *BRCA1* and *BRCA2* germline mutations overall prevalence was 0.23% in the common population, 2.8% in the BC cases before diagnostic age 70 and 6.1% in the patients before diagnostic age 50 [4]. Male breast cancer (MBC) incidence is less than 0.001% and the penetrance of male *BRCA2* mutation carriers is 5–10% for BC [7]. Risk estimations vary across different studies, which may be the result of sampling differences, mutation detection techniques, ethnic groups, and other factors.

The Human Gene Mutation Database (HGMD) has recorded more than 1467 *BRCA2* mutations. Of these, the majority of the disease-causing mutations were small deletions or insertions (54.7%) which induced premature termination codons. In some specific populations or ethnic groups, mutations are more frequent due to founder effects which are particularly remarkable in Ashkenazi Jews.

Currently, genetic testing is routine for women of families with a BC history. The testing regimes in China are limited [5]. The present study identified a heterozygous *BRCA2* c.5722_5723del mutation in a large Han-Chinese family with BC.

## Methods

### Subjects

**Figure 1:**
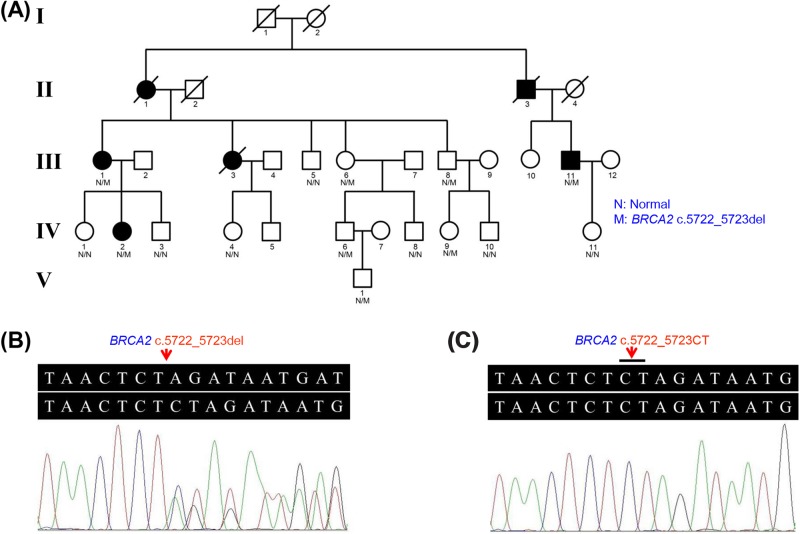
Pedigree of the family with BC and sequencing analysis of *BRCA2* c.5722_5723del mutation (**A**) Pedigree figure. Affected members are indicated by black symbols, and unaffected members are shown with white symbols. (**B**) Patient (III:1) with the heterozygous *BRCA2* c.5722_5723del allele. (**C**) Family member (III:5) with *BRCA2* c.5722_5723CT allele (absence of the c.5722_5723del).

The pedigree recruited for the present study consisted of 30 members in five generations of a Han-Chinese family with BC (Figure 1A). Peripheral blood specimens were obtained from 15 family members, including three BC patients (III:1, III:11, and IV:2) and 12 symptom-free members (III:5, III:6, III:8, IV:1, IV:3, IV:4, IV:6, IV:8, IV:9, IV:10, IV:11, and V:1). Written informed consent was obtained from each subject before participation. The study was performed in accordance with Declaration of Helsinki.

### Exome capture

Genomic DNA (gDNA) was isolated from peripheral blood by the phenol–chloroform method [8]. Whole exome sequencing (WES) of four family members, two typical BC patients (III:1 and IV:2) and two symptom-free members (III:5 and IV:1), was carried out to screen the known or unknown pathogenic gene mutation by BGI-Shenzhen. Qualified gDNA specimens were randomly broken into 150–250 bp by Covaris technology. These were subsequently executed to end-repairing, A-tailing, and adaptor ligation. Ligation-mediated PCR (LM-PCR) amplified the DNA fragments that were further purified and hybridized to the exome array for enrichment. Captured LM-PCR products were treated to generate DNA nanoballs using rolling circle amplification. After DNA quality assessment, the captured DNA library underwent high-throughput sequencing on BGISEQ-500 sequencing platform.

### Variant analysis

Raw data acquired from the BGISEQ-500 sequencing system was filtered to obtain clean reads. Burrows-Wheeler Aligner (BWA v0.7.15) was performed to align all clean reads to the human reference genome (GRCh37/hg19) [9]. HaplotypeCaller in the Genome Analysis Toolkit (GATK v3.3.0) is a reliable software for precise variant calling including single nucleotide polymorphisms (SNPs) and insertions/deletions (indels). Picard was used to remove duplicate reads. Local realignment and base quality score recalibration were carried out by GATK. Raw variation set including SNPs and indels was executed with hard-filtering method to obtain the highest quality call set for downstream analyses. SnpEff annotated SNPs and indels. Candidate variants were filtered against the 1000 Genomes Project, the NHLBI Exome Sequencing Project (ESP) 6500 database and in-house exome databases of BGI-Shenzhen. Sorting Intolerant from Tolerant (SIFT), Polymorphism Phenotyping version 2 (PolyPhen-2), MutationAssessor (MA), Condel, and Functional Analysis through Hidden Markov Models (FATHMM) were used to predict whether variants affected protein structure and function.

PCR products were separated via polyacrylamide gel electrophoresis (PAGE) and recovered from the crushed gel slice. Sanger sequencing, which relied on the recovered products, was used to validate putative disease-causing variants in the family using ABI3500 sequencer (Applied Biosystems Inc., Foster City, CA, U.S.A.) [10]. The primer sequences, designed by Primer3 [11], for locus-specific PCR amplification and Sanger sequencing were: 5′-TCAAAAATTTGCCAAACGAA-3′ and 5′-GAAACTTTCTCCAATCCAGACA-3′.

MutationTaster was applied to predict mutation pathogenicity.

## Results

### Clinical findings

**Figure 2:**
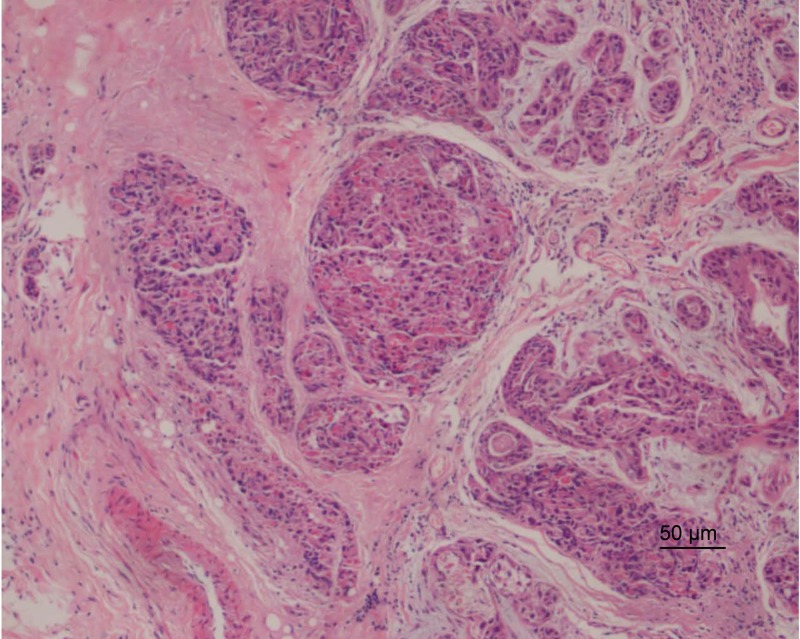
Pathological imaging of the patient’s (III:1) left breast lesions

The first symptom of BC is commonly a hard lump in the breast. Most BC is diagnosed via mammogram, ultrasound, or a biopsy of a sample from the affected area. The initial symptom of patients in the pedigree (III:1, III:11, and IV:2) was a lump in the left breast area. Pathological biopsy diagnosis indicated ductal carcinoma (Figure 2). Two affected family members (III:1 and IV:2) underwent surgical treatment. Detailed clinical data appears in Table 1.

**Table 1:** Clinical and genetic characteristics of family members with the *BRCA2* c.5722_5723del variant

Case	III:1	III:11	IV:2	III:6	III:8	IV:6	IV:9	V:1
Sex	Female	Male	Female	Female	Male	Male	Female	Male
Nucleotide change	c.5722_5723del	c.5722_5723del	c.5722_5723del	c.5722_5723del	c.5722_5723del	c.5722_5723del	c.5722_5723del	c.5722_5723del
Amino acid change	p. (Leu1908Argfs*2)	p. (Leu1908Argfs*2)	p. (Leu1908Argfs*2)	p. (Leu1908Argfs*2)	p. (Leu1908Argfs*2)	p. (Leu1908Argfs*2)	p. (Leu1908Argfs*2)	p. (Leu1908Argfs*2)
Zygosity	Heterozygous	Heterozygous	Heterozygous	Heterozygous	Heterozygous	Heterozygous	Heterozygous	Heterozygous
Age (years)	67	44	44	56	54	27	33	3
Age at diagnosis (years)	60	44	34	-	-	-	-	-
Symptom of onset	A lump in the left breast area	A lump in the left breast area	A lump in the left breast area	No	No	No	No	No
Pathological features	Ductal carcinoma	Ductal carcinoma	Ductal carcinoma	No	No	No	No	No

### Exome sequencing

On average, WES generated 192218706.25 effective reads for the four subjects, and 99.87% was mapped to the reference genome. Average sequencing depth on target was 221.13. The region was 99.49% covered at 10× or greater by the target sequence. There were – on average – 100383 SNPs and 16359 indels identified per individual.

### Identification of pathogenic mutation

Variants recorded in the 1000 Genomes Project with minor allele frequency (MAF) ≥1% and NHLBI-ESP6500 with MAF ≥1% were removed. Variants recorded in the in-house exome databases of BGI-Shenzhen were removed. SIFT (predicted score ≤0.05), PolyPhen-2 (output value ≥0.909), MA (predicted score >1.9), Condel (prediction result = deleterious), and FATHMM (prediction result = deleterious) indicated that the variant may be a potential disease-causing mutation. By WES, a heterozygous c.5722_5723del variant in *BRCA2* exon 11 was identified in the two patients (III:1 and IV:2), but absent in two unaffected individuals (III:5 and IV:1), confirmed by Sanger sequencing (Figure 1B,C). No other potential pathogenic mutations for BC were found. Sanger sequencing confirmed that the variant identified in the two patients was found in another patient (III:11) and five unaffected cases (III:6, III:8, IV:6, IV:9, and V:1). It was absent from other unaffected family members (IV:3, IV:4, IV:8, IV:10, and IV:11). MutationTaster suggested that the *BRCA2* c.5722_5723del variant was a disease-causing mutation. This variant was listed in ClinVar (http://www.ncbi.nlm.nih.gov/clinvar/) as ‘pathogenic’. According to the BRCA Share™ databases, the c.5722_5723del mutation was classified as ‘pathogenic’ [12].

## Discussion

In 1994, *BRCA2* was mapped to chromosome 13q12-q13 by linkage analysis of 15 *BRCA1*-unlinked families with early-onset BC [13]. Six different *BRCA2* germline mutations were reported in BC families 15 months later [14]. Subsequently, various *BRCA2* mutations such as missense, nonsense, frameshift, and splice site mutations were reported in BC patients worldwide [15].

In the present study, a heterozygous *BRCA2* c.5722_5723del mutation was identified in a five-generation Han-Chinese family having BC. The mutation was previously reported in BC and/or ovarian cancer patients from Hong Kong (China), Korea, and America [16–18].

The human *BRCA2* gene is a tumor suppressor which contains 27 exons and spans about 70 kb [19]. It encodes a protein consisting of 3418 amino acid residues. The exon 11 encodes more than 50% of the entire protein [20]. The BRCA2 protein plays a major role in homologous recombination (HR) that primarily repairs replication-associated DNA double-strand breaks (DSBs) during the S and G2 phases of the cell cycle [21]. It is a 390 kDa nuclear protein with four major domains: N-terminus, DNA binding domain (DBD), eight interspersed BRC motifs, and C-terminal domain [20,22,23]. The N-terminus binds to the coiled-coil domain of the partner and localizer of BRCA2 protein (PALB2), which is another protein related to BC susceptibility [20,22]. The DBD includes an α-helical structure, three oligonucleotide/oligosaccharide binding (OB) fold modules, and a tower domain (TD) [21]. The α-helical structure, OB1 and OB2 can bind with deleted in split-hand/split-foot 1 (DSS1) which associates with BRCA2 protein stabilization, facilitates HR, and participates in DNA repair, development as well as protein degradation [20,21]. The BRC motifs can also bind with RAD51. BRCA2-mutated cells are defective in repairing DSBs by HR. Heterozygous *BRCA2* mutations and loss of heterozygosity (LOH) associated haploinsufficiency of BRCA2 activity were proposed to promote BOC tumorigenesis [21,24]. Loss of wild-type *BRCA2* allele is not required for BC. Ovarian tumorigenesis is more dependent upon the loss of the wild-type *BRCA2* allele [24]. The *BRCA2* c.5722_5723del mutation is located in exon 11 and results in a frameshift after codon 1908 with a premature termination at codon 1909 (p.(Leu1908Argfs*2)). This may result in BC by disturbing HR. Mice with *Brca2* mutation suggested that the *BRCA2*-BC turmorigenesis depends more on homology-directed repair in the proliferating mammary gland than a specific reliance on BRCA2 [25].

The penetrance of the *BRCA2* c.5722_5723del mutation is 50% (2/4) in females and 25% (1/4) in males in the subject pedigree. This is consistent with the above-noted fact that *BRCA2*-related patients have different cancer risks at different ages [4]. In addition to genetic factors, other attributions for BC include reproductive and hormonal factors, prior health conditions, psychological states, lifestyles, and biological and environmental factors [1,26,27].

Genetic testing for disease-causing mutations in *BRCA1, BRCA2, PALB2*, and other BC susceptibility genes is strongly recommended for people with a BC and/or ovarian cancer family history. This report on a heterozygous *BRCA2* c.5722_5723del mutation in a large Han-Chinese family with BC may assist family members to mitigate BC risk factors. The study also supplements and improves genetic testing strategies and BC risk estimation methodologies for China. Additional functional analysis of the BRCA2 protein with this mutation is recommended and may result in additional information about the pathogenetic mechanism of BC.

## Abbreviations

BC: breast cancer
BOC: breast-ovarian cancer
*BRCA1*: the breast cancer 1 gene
*BRCA2*: the breast cancer 2 gene
DBD: DNA binding domain
DSB: double-strand break
FATHMM: Functional Analysis through Hidden Markov Models
GATK: Genome Analysis Toolkit
gDNA: genomic DNA
HR: homologous recombination
indel: insertion/deletion
LM-PCR: ligation-mediated PCR
MA: MutationAssessor
PALB2: partner and localizer of BRCA2 protein
PolyPhen-2: Polymorphism Phenotyping version 2
SIFT: Sorting Intolerant from Tolerant
WES: whole exome sequencing
